# Expanding the genetic code: phage-driven evolution of pyrrolysyl-synthetase for site-specific incorporation of synthetic phenylalanine and tyrosine derivatives

**DOI:** 10.3389/fmolb.2026.1737987

**Published:** 2026-03-17

**Authors:** Anastasia Dakhnevich, Sabina Miasoutova, Danila Iliushin, Aleksey Rozanov, Roman Ivanov

**Affiliations:** 1 Biotechnology Department, Sirius University of Science and Technology, Sirius, Russia; 2 Scientific Center of Genetics and Life Sciences, Sirius University of Science and Technology, Sirius, Russia

**Keywords:** directed evolution, noncanonical amino acid, PANCE, PylRS, pyrrolysyl-tRNA synthetase, tRNA

## Abstract

**Introduction:**

Pyrrolysyl-tRNA synthetase (PylRS) is a key enzyme for the site-specific incorporation of non-canonical amino acids (ncAAs) into proteins. However, its native form has a limited substrate scope. This study aimed to evolve PylRS from *Methanosarcina mazei* to enhance recognition and incorporation of tyrosine and phenylalanine derivatives.

**Methods:**

We used phage-assisted non-continuous evolution (PANCE) to generate a library of PylRS variants under selective pressure for the target ncAAs. Evolved variants were sequenced to identify mutations. Their aminoacylation efficiency and specificity were quantitatively assessed using fluorescence-based incorporation assays and mass spectrometry.

**Results:**

Sequencing revealed a polymorphic population of mutations, with a significant cluster located within the enzyme's amino acid binding pocket. Several evolved variants showed an orders-of-magnitude increase in the efficiency of incorporating their target ncAAs compared to the wild-type enzyme.

**Discussion/Conclusion:**

Our findings confirm that PANCE is a highly effective method for engineering PylRS variants with strong and specific activity towards desired non-canonical amino acids. The identified mutations, particularly those in the binding pocket, provide a basis for understanding and further optimising substrate specificity in synthetic biology applications.

## 1 Introduction

In living systems, proteins represent the main class of functionally active molecules. They act as catalysers, substrates and enzymes for biochemical reactions (Appell et al., 2018). The number of monomers required to build proteins is limited in nature to only twenty standard amino acids and two additional ones: selenocysteine (Sec) and pyrrolysine (Pyl), which are found in only a few species (Wan et al., 2014; Wright and O’Donoghue, 2023). At the same time, there are over a thousand non-proteinogenic amino acids (ncAA) whose incorporation into proteins can improve their properties, such as stability or activity (Narancic et al., 2019).

It is important to understand that both non-canonical amino acids require specialised translation mechanisms. For Sec, this element is a specialised regulatory sequence and enzymes that modify serine, while the translation apparatus for Pyl is represented by aminoacyl-tRNA synthetase (aaRS) and tRNA pyrrolysine (tRNA_Pyl_), which is a natural example of genetic code extension (GCE) (Mariotti et al., 2013; Gong et al., 2023; Dakhnevich et al., 2025). To realise GCE under laboratory conditions, modern biotechnology offers two main approaches: the reassignment of sense codons and the site-specific incorporation of ncAA in response to a stop codon or a non-triplet codon. The first approach involves the incorporation of ncAAs that have structural similarities to natural amino acids using native aaRSs (Melnikov and Söll, 2019; Andrews et al., 2023). The second approach requires the presence of a free codon and exogenous aaRS/tRNA pairs (Ding W. et al., 2020). The stop codon, termed “amber,” was selected due to its low abundance. In *Escherichia coli*, the amber codon is involved in the termination of translation of proteins that are often not essential for cellular metabolism. This avoids negative effects and the possible formation of toxic proteins (Chatterjee et al., 2012).

aaRSs that do not interact with proteinogenic amino acids and endogenous tRNAs, but have the ability to participate in protein synthesis by selectively adding a non-proteinogenic amino acid to the translation product, are termed orthogonal (de la Torre and Chin, 2021). One of the most effective and widely used translation systems to implement this method is pyrrolizyl-tRNA synthetase (PylRS), which aminoacylates its cognate tRNA (Srinivasan et al., 2002; Fischer et al., 2022). They are initially orthogonal to the endogenous translation systems of the host organism (Wang et al., 2001; Rogerson et al., 2015; Krahn et al., 2020), have a large amino acid binding pocket in the active site and lack an editing domain, which is important for the ability to recognise a large number of ncAAs (Kavran et al., 2007; Yanagisawa et al., 2008). At the same time, tRNA_Pyl_ is a suppressor of the amber codon, and the anticodon is not an identity element for PylRS, so this system can be used to recode the UAG codon (Suzuki et al., 2017). However, the optimisation of synthetases to increase their efficiency, both in terms of selectivity and biochemical properties, is crucial for the further development of the technology. The production of aaRS specific for certain ncAAs is possible by rational engineering or directed evolution methods such as phage-associated evolution (PACE) (Ho et al., 2021).

Between 2017 and 2020, the PACE method was developed and successfully adapted for use with aaRS (Esvelt et al., 2011; Bryson et al., 2017; Ho et al., 2021). However, there is a technically simplified version, PANCE (phage-assisted non-continuous evolution) (Ho et al., 2021). This approach is a discontinuous version of PACE in which mutagenesis and selection cycles are performed manually by re-infecting bacteriophages from one sample to the next. No complex equipment is required, which makes PANCE accessible to a larger number of laboratories, but the implementation is more labour-intensive and time-consuming (Esvelt et al., 2011; Ho et al., 2021).

The creation of new aaRSs makes it possible to incorporate amino acids not found in nature into proteins, increasing the diversity of chemical groups, modelling biological processes and conferring unique properties to proteins (Beattie et al., 2023). Until recently, ncAAs were only incorporated into proteins and peptides by chemical synthesis (Evers et al., 2017). This process is much more expensive than the production of recombinant proteins in bacterial and mammalian cells (Zheng et al., 2025). Therefore, the development of recombinant protein and peptides with ncAAs is an urgent technological challenge that requires extensive fundamental research (Ding Y. et al., 2020; Song et al., 2021).

In this study, we performed directed evolution of native PylRS and a chimeric construct based on domains from *Methanosarcina barkeri* and *Methanosarcina mazei* to increase the activity and specificity for four ncAA derivatives of phenylalanine and tyrosine. We used the PANCE method, which involves four stages of increasing selection pressure and alternating positive and negative selection. During evolution, key mutations associated with improved tRNA binding and enzymatic activity were identified. Functional assessment of the mutants using the sfGFP fluorescence system revealed a significant increase in activity compared to the original variant. This enables us to develop unique, highly efficient synthetases for the targeted incorporation of amino acid derivatives into proteins, which are promising for biopharmaceuticals and fundamental research.

## 2 Materials and methods

### 2.1 Strains and plasmid constructs

Antibiotics (Gold Biotechnology) were used to prepare the selective media in the following working concentrations: carbenicillin – 50 μg/mL; spectinomycin – 50 μg/mL; chloramphenicol – 30 μg/mL; kanamycin, 30 μg/mL; tetracycline – 15 μg/mL; streptomycin – 50 μg/mL.

All oligonucleotides were synthesised at the Genetic Engineering Core Facility of Sirius University using an ASM-800ETf DNA/RNA synthesiser. PCR was performed using the Q5 Hot Start High-Fidelity DNA Polymerase (New England Biolabs). Plasmids and selection phages were prepared by isothermal assembly with Gibson Assembly 2x Master Mix (New England Biolabs), SLIC with T4 DNA polymerase (SybEnzyme, Russia) or Golden Gate Assembly with SapI/BspQI restriction enzymes (New England Biolabs).

The plasmid pDB007ns2a-neg was created from two donor plasmids: pDB007ns2a (Addgene #99214) and pDB023f. The original plasmids were digested with the restriction enzymes NcoI and XbaI (SibEnzyme, Russia). When creating the plasmid pDB016-tRNA_Pyl_, the donor fragments were pDB016 (Addgene #99220) and pDB038. The tRNA_Pyl_ gene was amplified from pDB038 using primers with NcoI restriction sites at both ends. The plasmid pDB016 and the amplicons containing the tRNA_Pyl_ gene were digested with the restriction enzyme NcoI (SibEnzyme, Russia). Both assemblies were performed with T4 DNA ligase (SybEnzyme, Russia).

The pHPyl-PylRS-GlnS plasmid was assembled from two fragments: one containing the aaRS gene with promoter and terminator extended with long oligonucleotides at the ends of the amplicon, and the second containing the origin of replication and regulatory region as well as the chloramphenicol resistance gene. Plasmid assembly was performed using the Gibson Assembly Reagent (New England Biolabs, United States) according to the manufacturer’s protocol.

Another plasmid, pET-sfGFP-27TAG, contains the sfGFP sequence with an amber codon at position F27. The plasmid was assembled from three fragments: The first contains the sfGFP sequence, the second contains the origin of replication and regulatory sequences, and the third contains kanamycin resistance. All three fragments were amplified from different plasmids. The primers were designed so that the amplicons contained SapI restriction sites at both ends. The assembly was performed according to the Golden Gate method using the SapI enzyme (New England Biolabs, United States). The plasmid was incubated overnight at 37 °C.

All plasmid vectors used in this study are listed in Supplementary Table S1.

Plasmid DNA was isolated using the Qiagen Spin Miniprep Kit according to the manufacturer’s instructions. All constructs were verified by Sanger sequencing on an Applied Biosystems 3730xl DNA analyser (48 capillaries, 50 cm) (Thermo Scientific™, United States) using POP-7 polymer.

The following aromatic ncAAs were used in this study: O-methyl-L-tyrosine, 4-azido-L-phenylalanine (Tokyo Chemical Industry Co., Ltd.), O-allyl-L-tyrosine (synthesised from O-benzyl-L-tyrosine at the Medicinal Chemistry Core Facility of Sirius University) and 2-chloro-L-phenylalanine (Shanghai Acmec Biochemical Co., China).

### 2.2 Phage libraries

The filamentous bacteriophage M13 was selected for the implementation of PACE. The sequence encoding pIII was replaced by gene of interest (GOI). GOI is an aaRS sequence specific to the archaeon *M. mazei*. A ChPylRS gene was also created in which the N-domain of *M. barkeri* ― MbPylRS - and the C-domain of *M. mazei* - MmPylRS - were adopted, with a joining sequence between the domains. The mutations N346A/C348A were introduced into the C-domain of both synthetases (Wang et al., 2013). The phages were assembled from three fragments: pBT29-splitC, pBT29-splitD (depositor David Liu, Addgene #122598) and the synthetase gene using the Golden Gate method at the SapI restriction site.

Phage library titres were determined using either two qPCR methods or a plaque assay using *E. coli* S2060 (Addgene #105064) with the pJC175e plasmid (Addgene #79219) to produce phage particles. qPCR was performed according to the following protocol: phages with a known titre of 1 × 10^10^ PFU/mL were serially diluted tenfold at a 1:10 ratio to create a calibration curve. qPCR was then performed using the diluted samples and a positive control wild-type bacteriophage (titre of 1 × 10^6^ PFU/mL). The resulting calibration curves enabled correlation of the cycle number and titre of the bacteriophage in the control sample with the titre of the phages obtained during PANCE. 1 μL of the cell suspension containing phages of unknown titre was added to 24 μL of the reaction mixture for RT-PCR with the required primers (SplitC_gV_for_qPCR_F 5′-CACCGTTCATCTGTCCTCTTTCAA-3′; SplitC_gV_for_qPCR_R 5′-CGACCTGCTCCATGTTACTTAGC-3′; SplitC_gV_for_qPCR_probe 5′-FAM-CATAAGGGAACCGAACTGACCAAC-BHQ1-3′) and HS-qPCR (2×) (Biolabmix, Russia). The annealing temperature of the primers was 60 °C, the elongation time was 15 s. A total of 40 cycles were performed (Miller et al., 2020).

### 2.3 PANCE

Positive selection was performed using *E. coli* S2060 cells (Addgene #105064). For the first PACE step, the cell system contained the plasmids pDB023f (Addgene #99217) with two amber codons in the T7 RNA polymerase gene and pDB021CH(+) (Addgene #99209) with gIII under the T7 promoter. For the second PACE step, the plasmid pDB038 (Addgene #99210) containing the gIII gene with a stop codon was used. The plasmid pDB038a (Addgene #99210), which is intended for the third step of positive selection, has two stop codons. Plasmid pDB038b (Addgene #99210) contains three stop codons and was used in the fourth round of positive selection. The pMP6C (MP) mutagenesis plasmid was used in all positive selection rounds.

Positive selection rounds were performed in 2XYT medium supplemented with 2–10 mM ncAA and the required antibiotics. The upper limit of ncAA concentration is 5 mM for O-Allyl-L-Tyr and 10 mM for the other selected amino acids due to their low solubility in aqueous solutions. To dissolve the ncAA in 2xYT medium, an ultrasonic bath was used while maintaining the temperature below 30 °C. Cells containing the plasmids for each round were grown at 37 °C until an OD600 of 0.5–0.6 was reached. 5 mM arabinose was added to the cell culture to induce mutagenesis, followed by transfection with a minimal inoculum of 10^6^ PFU/mL of the selection phage from the previous PANCE cycle (Suzuki et al., 2017). The infection was incubated overnight at 37 °C. Prior to the next infection, the cells were centrifuged at 8,000 rcf for 5 min, then the supernatant was collected and filtered with a 0.22 μm filter. qPCR was used to update bacteriophage titres after each stage.

Negative selection was performed with the plasmid pDB016+tRNAPyl, which contained a mutated gIII-neg gene with a deletion. The pDB007nsa2-neg plasmid, which contained the gIII gene without stop codons under the phage shock promoter and T7 RNAP with stop codons, was also used for negative selection. Infection for negative selection was carried out in 2xYT medium with the necessary antibiotics, without ncAA. The cells were grown to an optical density at 600 nm (OD600) of 0.5–0.6. Infection was performed with a sample of the supernatant of the previous infection containing 10^6^ PFU/mL of the selective phage. The infection mixture was incubated for a maximum of 4 h.

### 2.4 MiSeq

The main protocol for creating the gene library, AmpliSeq, is described in the corresponding article (Cruaud et al., 2017). A total of eight primer pairs were used for gene amplification, designed to cover the entire gene length twice. The final library quality was assessed by capillary electrophoresis using an Agilent TapeStation 4150 (Agilent Technologies, United States). The library concentration was greater than 20 ng, which met the requirements for loading onto the Illumina MiSeq platform. Sequencing with the Illumina MiSeq was performed at the Genomic Research Core Facility of Sirius University.

The first step in processing the sequencing results was to clean the data of short reads (less than 50 nucleotides) and remove adapter sequences using Trimmomatic v0.39. Read quality before and after filtering was assessed using FastQC v0.12.1. The filtered reads were aligned to the reference sequence of the PylRS gene using Bowtie2 v2.4, with unaligned reads removed. Coverage analysis included estimating depth using the samtools depth command and checking the insert size distribution using Picard CollectInsertSizeMetrics.

Genetic variants were identified using FreeBayes v1.3.9 with the following parameters: alignment quality of at least 20, minimum coverage of 10x and alternative allele frequency of at least 1%. The final consensus sequences were generated in bcftools v1.9 with bcftools consensus. Visual inspection of alignments and detected mutations was performed in IGV (Integrative Genomics Viewer v2.12).

For adapter trimming, the ILLUMINACLIP parameter with the NexteraPE-PE.fa library was used in Trimmomatic (seed mismatches = 2, palindrome clip threshold = 30, simple clip threshold = 10). Quality filtering included trimming bases with a quality score below five from the ends of reads (LEADING:5, TRAILING:5), as well as processing with a 4 bp sliding window requiring an average quality of at least 15 (SLIDINGWINDOW:4:15). Minimum base quality was set to 20, and the minimum alternate count was set to 10, indicating the minimum number of reads with an alternative allele.

### 2.5 *In vivo* fluorescence assays

The aaRS genes were obtained by PCR with Fusion DNA polymerase (Thermo Scientific™, United States), the bacteriophage pool obtained after the fourth round of PACE was used as a template. A fragment from the pHPyl-PylRS-GlnS plasmid was used as the vector. The pHpyl-PylRS-GlnS plasmid containing the aaRS of interest and pET-sfGFP-27TAG were cotransformed into electrocompetent BL21 Star cells. Cells were reconstituted in SOC for 1 h with shaking at 37 °C and then plated out and grown overnight at 37 °C on LB agar with kanamycin and chloramphenicol. Individual colonies were inoculated with 800 µL 2xYT medium containing antibiotics and grown overnight at 37 °C with shaking at 750 rpm. A 96-well deep-well plate was prepared for the assay. 760 μL of medium, either with or without ncAA (2 mM), was added to each well and 40 µL of the saturated overnight culture was transferred. Each sample was added in triplicate. Expression was induced by adding IPTG at a final concentration of 1 mM. Cultures were grown at 750 rpm and 37 °C. A 200 µL aliquot of each culture was transferred to 96-well flat-bottomed Costar plates and fluorescence (λex = 485 nm, λem = 528 nm) and optical density (OD) were measured using a CLARIOstar Plus (BMG Labtech, Germany) plate reader. Normalisation was performed solely to the optical density of the culture (OD600), and background subtraction was carried out for the respective cultures, with and without the addition of ncAA respectively.

Cells natively expressing sfGFP (the gene with no amber codons) were used as fluorescent controls, while clones containing the sfGFP plasmid with amber codons and the wild-type PylRS plasmid and pure medium served as negative controls.

Data visualisation and statistical analysis were conducted using GraphPad Prism version 8.4.3 (United States). The normality of the distribution of quantitative indicators was assessed using the Kolmogorov-Smirnov and Shapiro-Wilk tests. The non-parametric Mann–Whitney test was used to determine the statistical significance of differences between independent quantitative samples that did not follow a normal distribution. Differences were considered statistically significant at a p-value less than 0.05. The sample comprised three technical replicates of the fluorescent ncAA incorporation test for one mutated PylRS clone.

### 2.6 Protein expression and purification

The pET-sfGFP-TAG plasmid was co-transformed with different PylRS variants in *E. coli* BL21 Star. After 1 hour of recovery, the bacteria were spread on a plate with kanamycin and chloramphenicol. A single colony was removed from the plate and cultured overnight in 1 mL 2xYT medium. The cultured bacteria were then transferred to 50 mL of fresh medium containing the required antibiotics and 2 mM of the appropriate ncAA and incubated at 37 °C until an OD600 of 0.6–0.8 was reached. Protein expression was induced by addition of 1 mM IPTG and incubated at 37 °C for 16–18 h. The cells were centrifuged at 6,000 rpm for 10 min. Centrifugation was repeated until the entire cell culture pellet was obtained. The mass of the cell pellet was measured and then resuspended in buffer A (50 mM of Tris-HCl, 0.0005 M imidazole and 0.3 M NaCl, pH 7.8) at a ratio of 1:3 pellet mass to buffer mass.

Homogenisation was performed with a UC-650 Ultrasonic Cell Disruptor (MIULAB, China) using sonication cycles of 5 s on, 5 s off for 15–35 min. Samples were then centrifuged again at 17,000 rpm for 10 min and the supernatant was collected and filtered through a 0.22 μm filter. sfGFP was purified by affinity chromatography on a Ni-containing BioRad Nuvia IMAC column. Elution was performed with buffer B (50 mM of Tris-HCl, 500 мМ imidazole, pH 7.5).

### 2.7 Mass spectrometry

Electrospray ionisation mass spectrometric characterisation of sfGFP was carried out at the Analytical Core Facility of Sirius University. HPLC-MS analysis of samples was performed using a Bruker maXis II 4G ETD high-resolution quadrupole time-of-flight mass spectrometer (Bruker, Billerica, MA, United States) with an electrospray ionisation (ESI) and collision-induced dissociation (CID) source, coupled to a Thermo Scientific UltiMate 3000 ultra-high-performance liquid chromatography system (Thermo Scientific™, United States) equipped with a binary gradient pump, degassing module, automatic thermostatic injector, and column thermostat.

Before analysis, the mass spectrometer was calibrated according to the manufacturer’s recommendations. The analysis was conducted using a Waters BioResolve RP chromatographic column, 2.7 µm, 2.1 mm × 50 mm, in reversed-phase elution mode. Mobile phase A was water with 0.1% formic acid and 0.02% TFA; mobile phase B was acetonitrile with 0.1% formic acid. The column temperature was maintained at 60 °C. Each sample, containing 10 ng, was diluted fivefold with mobile phase A. The chromatographic gradient and flow rate are described in the Table 1.

**TABLE 1:** The chromatographic gradient and flow rate.

Time, min	Mobile phase А, %	Mobile phase В, %	Speed, mL/Min
0.0	95	5	1.2
1.0	95	5	1.2
1.7	75	25	0.4
6.4	50	50	0.4
7.0	5	95	1.2
7.7	5	95	1.2
8.0	95	5	1.2
10.0	95	5	1.2

The injection sequence included a solution of the IgGk reference material NIST 8671, all samples, and a blank solution (mobile phase A). Data was collected by the mass spectrometer in scanning mode over the range 800–7,000 m/z. An ESI ionisation source was used in positive ionisation mode. The acquired data were processed using Byos® Protein Metrics software (Byos, Boston, MA, United States). System suitability was assessed against acceptance criteria, deconvolution was performed, theoretical and measured masses were compared, and the relative abundances of all detected protein isoforms were estimated.

### 2.8 Modeling of protein structures

Sequence alignment and mutation search in ch PylRS variants were carried out using the MEGA (Molecular, Evolutionary, and Genetic Analysis) programme. Structural analogues were identified using the PDB (Protein Data Bank) database. The most similar crystal structures were selected as templates for the study: for the N-domain, the 5V6X structure, which has 87% sequence identity to the reference sequence (Seq Identity = 87%); for the C-domain, the 2ZCE structure (Seq Identity = 99%). The search was based on the reference amino acid residues of the N- and C-domains of PylRS.

Three-dimensional models of the mutant enzymes were constructed using the SWISS-MODEL online service. The quality of the constructed models was assessed using the Global Model Quality Estimation (GMQE) parameter: GMQE = 0.79 for the N-domain and 0.82 for the C-domain, indicating high quality of the initial determination. Spatial structure analysis, model visualisation, and point defect introduction were performed using PyMOL version 3.1.5.1.

## 3 Results

### 3.1 Construction of the MmPylRS(mut) and СhPylRS(mut) genes

The most commonly used enzymes for ncAA incorporation are pyrrolizyl-tRNA synthetases isolated from *M. mazei* (MmPylRS) and *M. barkeri* (MbPylRS). Due to its greater stability and activity (Wang et al., 2013; Dakhnevich et al., 2025), MmPylRS was chosen as one of the target proteins for directed evolution. The codon composition of the gene was optimised for more efficient expression in *Escherichia coli* cells.

**FIGURE 1:**
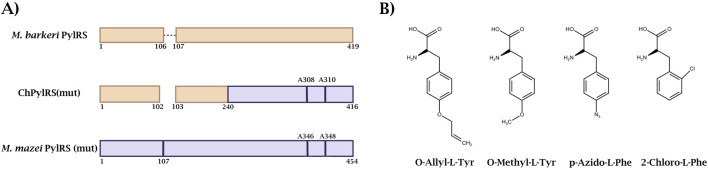
**(A)** The structure of PylRS with introduced mutations used in this study **(B)** Structures of ncAAs used in this study.

To create a PylRS variant with increased efficiency for incorporating n-amino acids containing a benzene ring for further PANCE, a double mutant of the enzyme with Asn346Ala and Cys348Ala substitutions (N346A/C348A) was used as the basis. These mutations were selected because they are located in the enzyme’s active site, in the region responsible for binding the amino acid substrate. These substitutions are known to be critical for expanding the substrate specificity of PylRS toward various phenylalanine derivatives bearing bulky substituents, such as alkyne, azide, ketone, or halogenated groups, at the meta- and ortho-positions of the benzene ring (Wang et al., 2013; Tharp et al., 2014). The structural basis for this effect is an increase in the volume and hydrophobicity of the amino acid binding pocket, which creates space for specificity toward larger and branched substrates. However, despite expanding the spectrum of recognized amino acids, this mutant does not provide high selectivity or catalytic efficiency for the specific target ncAA. To further direct evolution, the MmPylRS gene encoding this double mutant (MmPylRSmut) was synthesized *de novo* and cloned into an appropriate vector system (Figure 1A).

A literature search revealed a variant of the chimeric ChPylRS protein in which the N-domain is from *M. barkeri*, MbPylRS, and the C-domain is from *M. mazei* MmPylRS, with a gap between the domains (Bryson et al., 2017). A schematic representation of the ChPylRS(mut) gene is shown in Figure 1A. This PylRS variant has a higher activity towards pyrrolysine and a higher solubility, which distinguishes it favourably from the wild-type enzyme. It is also interesting that a similar structure with two effector domains and a gap between them belongs to the “PylSn+PylSc” class (Fladischer et al., 2019). In this class, the domains are expressed from two separate genes as two different proteins, which are then assembled into PylRS (Guo et al., 2022). We adopted this chimeric PylRS as a starting scaffold for directed evolution in our proof-of-concept experiments to test our selection strategy and to yield highly active variants. In this way, two synthetase genes were obtained, which were further used in directed evolution experiments in relation to four derivatives of phenylalanine and tyrosine shown in Figure 1B.

MmPylRS(mut) was used in the experiment with O-Allyl-L-Tyr (AllY, AllYPylRS), O-Methyl-L-Tyr (MethY, MethYPylRS), 4-Azido-L-Phe (AzF, AzFPylRS), while ChPylRS(mut) underwent evolution in relation to 2-Chloro-Phe (2ClF, 2ClFPylRS).

### 3.2 Developing a PANCE protocol

**FIGURE 2:**
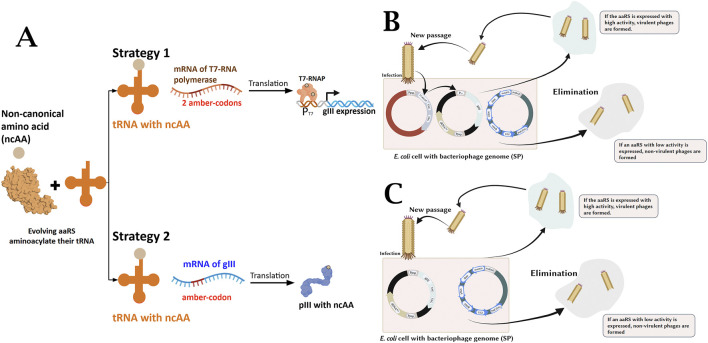
**(A)** Strategies for linking ARS activity to the expression of gIII, which encodes the pIII protein required for phage to be infectious. In strategy 1, ARS-catalyzed aminoacylation of tRNA_Pyl_ enables translation of full-length T7 RNAP from a transcript containing amber stop codons. T7 RNAP subsequently drives expression of gIII from the T7 promoter (PT7). In strategy 2, orthogonal aminoacylation permits full-length translation of pIII from gIII mRNA containing amber stop codon. **(B)** Schematic representation of PANCE experiment. Stage 1: Amber codons (red crosses) in T7RNAP, gene driven by phage shock promoter (psp); gIII expressed from T7 promoter. **(C)** Schematic representation of PANCE experiment. Stage 2: Single amber stop codon in gIII, gene expressed from psp. Stage 3: gIII with two amber codons, gene expressed from psp. Stage 4: gIII with three amber codons, gene expressed from psp.

For this study, a variant of the previously described PANCE system (Suzuki et al., 2017) was adapted. This approach exploits the life cycle features of M13 filamentous bacteriophages, in which the pIII protein plays a key role (Hay and Lithgow, 2019). This protein is encoded by the gIII gene and is involved in both the initial stages of infection and the final stages of virion assembly, release, and detachment from the cell membrane. To implement the PANCE method, the gIII gene in SP is replaced with the gene encoding the target protein PylRS. The host cells contain a helper plasmid (AP) with the gIII gene, the expression level of which depends on PylRS activity. Thus, bacteriophages carrying more active variants produce particles faster and replicate more efficiently, ensuring the selection of more active mutants of the gene of interest (Miller et al., 2020). In our system, gIII gene expression depends on the activity and selectivity of binding of the target amino acid to tRNA_Pyl_ by the PylRS enzyme (Figure 2A). Increasing the efficiency of PylRS with a new substrate increases the rate of pIII protein production and, consequently, the rate of phage replication.

For genes of interest with low initial activity, several rounds of positive selection with gradually increasing selective pressure are necessary. Selection pressure depends on the location and number of amber codons, the concentration of ncAA, and the bacteriophage culture time. During PANCE, we aimed to organise the process so that selection conditions were as stringent as possible, as excessively lenient conditions promote the accumulation of bacteriophages carrying synthetases with off-target activity, while excessively stringent conditions lead to the loss of the population of evolved bacteriophages that are unstable under the new conditions.

In the first stage of positive selection, we used *E. coli* S2060 cells transformed with two helper plasmids (APs). One contained two amber codons in the T7 RNA polymerase gene, while the gIII gene, necessary for the formation of virulent bacteriophages, was placed under the T7 promoter in the second AP with tRNA polymerase (Figure 2B). This approach increases pIII production, as each amber codon suppression event can result in multiple gene III transcripts. In the second stage, the cells were transformed with one AP containing a single amber stop codon in the gIII gene, as well as the tRNA_Pyl_ encoding sequence (Figure 2C). Phage replication depends on the successful aminoacylation of tRNA_Pyl_ by the selected PylRS and subsequent suppression of the TAG codon in gIII. At this stage, selection pressure is increased because one pIII is now produced by suppressing one stop codon. The concentration of the target amino acid in the medium at this stage was maintained at 10 mM (for all non-canonical amino acids except AllY, whose solubility threshold was 5 mM). At each subsequent stage, the selection conditions became increasingly stringent: the plasmid for the third PACE stage contained the gIII gene with two amber stop codons, while for the fourth stage it had three (Figure 2C). In addition, at the fourth PACE stage, we sequentially decreased the concentration of non-canonical amino acids (ncAA) from 10 mM at the beginning to 2 mM at the end, creating conditions for the selection of the most effective PylRS. Attempts were made to transfer bacteriophages to conditions with 1 mM of the studied ncAA. However, the results of several adaptation tests with decreasing amounts of ncAA showed that the most stable titer differences between the presence and absence of ncAA in the medium were observed at a final concentration of 2 mM. Thus, the positive selection stage is aimed at obtaining mutant variants of PylRS.

Successful directed evolution requires multiple rounds of PANCE to achieve stable target activity of the mutated protein. The system can also be adapted for negative selection to isolate bacteriophages carrying a gene with off-target activity. The initial stages of selection, known as positive selection, result in the formation of the most active aaRSs, while the subsequent stage, called negative selection, involves eliminating synthetases with non-specific activity. This stage requires a plasmid containing gIII-neg. Unlike positive selection, gIII-neg contains a mutant C domain with a 70-amino acid deletion, the absence of which leads to the formation of a phage particle incapable of catalysing its detachment from the host cell (Carlson et al., 2014). Due to the proven effectiveness and versatility of negative selection, this approach was chosen for the present study. However, previously described negative selection approaches are performed simultaneously with mutagenesis and involve inducing expression of a dominant-negative form of gIII in parallel with positive selection. This can lead to premature elimination of mutants with potentially beneficial activity, especially when ncAAs, which are required for mutagenesis during aaRS adaptation to a new substrate, are added to the medium. Due to these limitations, in this study, negative selection was performed manually as a separate step after each round of mutagenesis, without the addition of ncAAs.

**FIGURE 3:**
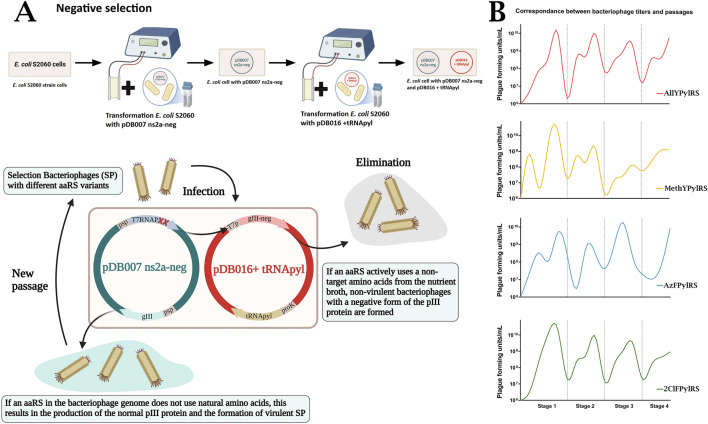
**(A)** Negative selection: Uses a plasmid with a deletion form of gIII (gIII-neg) without amber codons under, controlled by T7 promoter; T7RNAP under psp. Co-expressed with intact gIII under psp. These plasmids act together in host cells. **(B)** Summary of phage titers over the whole PANCE experiment, in the presence of ncAA. Titers are individual measurements based on the average of three technical replicates of the qPCR assay.

Two plasmids, pDB007ns2a-neg and pDB016+tRNAPyl, were successfully constructed for use in the PANCE process. Plasmid pDB007ns2a-neg contains an intact gIII gene and the T7 RNA polymerase gene under the control of the phage shock promoter, with two amber stop codons. In this system, when T7 RNA polymerase is activated (i.e., when PylRS inserts natural amino acids in place of the amber stop codon in the T7RNAP transcript), the level of gIII-neg production is sufficient to dominantly inhibit infectious phage assembly due to the presence of intact gIII under the control of the phage shock promoter. The activity of plasmid pDB007ns2a-neg is crucial, as it ensures the assembly of active viral particles that will be transmitted further along the evolutionary lineage. Without expression of the intact gIII gene, all SPs, including those with target activity, would be lost. Plasmid pDB016+tRNAPyl contains the gIII-neg gene under the T7 promoter and the tRNAPyl coding sequence. This gene is necessary to achieve a selective effect: when aaRSs are nonspecifically active, a variant of the pIII protein with a deletion is expressed, which prevents such SPs from actively reproducing in the evolving lineage. Working together, the pDB007ns2a-neg and pDB016+tRNAPyl plasmids maintain SP titre while eliminating unwanted aaRSs activity, without requiring positive selection plasmids in PANCE. Negative selection was performed without adding noncanonical amino acids to the cells. Thus, it is expected that aaRS variants capable of incorporating natural amino acid analogues will be excluded from the selection (Esvelt et al., 2011; Bryson et al., 2017). A schematic representation of the negative selection step is shown in Figure 3A.

Upon transition to the next stage of positive selection, phage adaptability to the current selection conditions was tested. Two cell cultures were prepared: one supplemented with the corresponding amino acid at a concentration of 2–10 mM, depending on the stage, and the other grown in 2xYT medium without ncAA. Differences in bacteriophage titres were assessed using qPCR results (Supplementary Figure S1). If titres differed by 1.5–2 orders of magnitude or more when ncAA was added to the medium compared to conditions without ncAA, the gene of interest was considered to have achieved sufficient specificity for further directed evolution and transition to the next stage. In other cases, several rounds of negative selection were performed, after which the test was repeated. During the more than 50 PANCE generations in our study, only for AzFPylRS did we have to return to the previous, less stringent selection conditions at the final stage of positive selection.

The optical density of cells infected with a new batch of bacteriophage was consistently 0.6. The cultivation time for the positive selection stages was approximately 16–18 h, while for negative selection it was a maximum of 4 h. When determining the optical density of cells, cultivation time, and the amount of bacteriophage for inoculation in pilot experiments, we followed the protocol described by Suzuki et al. (2017), maintaining the PFU level during each infection to control phage dissemination in the new cell culture (Figure 3B).

### 3.3 PANCE result

We conducted 50 generations of directed evolution and retained one independent lineage of evolved phages for each ncAA. The degree of adaptation of the mutated pylRS was assessed by the increase in bacteriophage titer. For example, during the third and fourth stages of evolution, a steady increase in titer was observed, reaching maximum values of approximately 10^8.5^–10^9.5^ PFU/mL at the end of the mutagenesis round. When conditions were changed to more stringent ones, the titer dropped sharply below 10^8^ PFU/mL, as shown in Figure 3B. It is important to note that titer growth does not reflect the functional specificity of pylRS but serves only as a general marker of enzyme activity retention in the evolutionary lineage. If the SP titer is not restored upon transition to the next PACE stage, this indicates the need to return to less stringent mutagenesis conditions.

After 12 rounds of selection with three TAG codons, a reduction in ncAA content in the medium, and an additional round of negative selection, we tested the evolving bacteriophages with and without the corresponding ncAAs. Phage titres showed that the evolved phage lineages replicated significantly more efficiently in the presence of their amino acids, indicating the emergence of more active PylRS variants (Supplementary Figure S1). During the PANCE protocol, we encountered several methodological challenges similar to those described by Miller et al. (2015). Employing a qualitative problem analysis enabled us to address these issues consistently. The most common challenge was poor bacteriophage adaptation after the negative selection step, which appeared as a statistically insignificant difference between phage titres in the presence of ncAA and in the control conditions without ncAA. However, in most cases, repeating several additional rounds of negative selection resulted in achieving a threshold titre difference sufficient to proceed to the next stage of the experiment.

**FIGURE 4:**
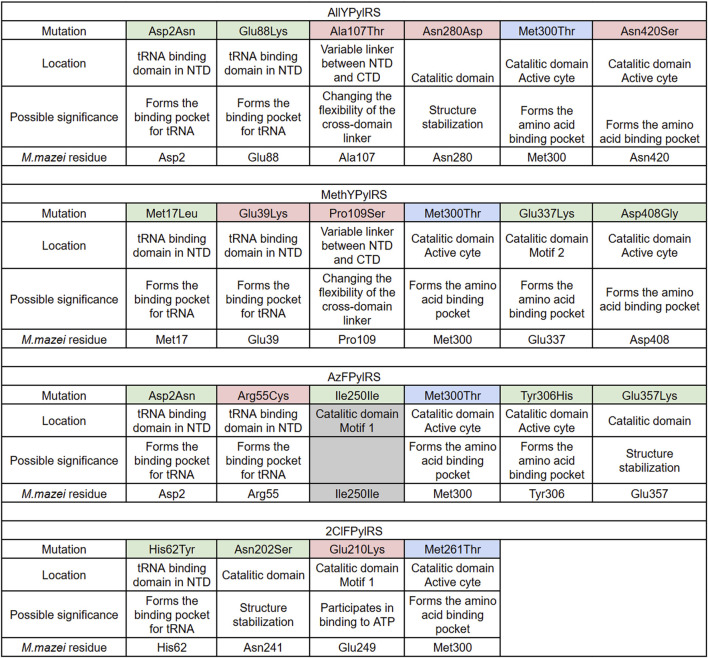
PylRS mutations resulting from PANCE and their locations in the protein as well as the corresponding residues in *Methanosarcina mazei*, determined by multiple sequence alignment. Mutations detected by Illumina MiSeq sequencing are highlighted in green. Mutations detected in the best clones by Sanger sequencing are highlighted in red. Mutations that occurred independently in all clones are highlighted in blue. Mutations that do not lead to amino acid substitutions are highlighted in grey.

To identify and compare the acquired mutations, amplicon-directed sequencing was performed on the Illumina MiSeq platform (Supplementary Table S2), and Sanger sequencing of individual clones was conducted after efficiency analysis for sfGFP. Consensus nucleotide sequences of the pylRS genes were obtained for each evolutionary lineage at each stage of mutagenesis. The previously introduced mutations Asn346Ala and Cys348Ala (Asn205A and C207A substitutions in the C-domain of chPylRS) were retained in all cases. Another notable observation was the Met300Thr mutation, which was independently fixed in all evolutionary lineages (Figure 4).

Evolution of aaRSs by the PANCE method using various noncanonical substrates resulted in the accumulation of a large number of mutations. All mutations that arose in our system are shown in Table 1. Most mutations occurred in the C-domain of aaRS. Mutations that exchange large amino acids for smaller ones near the active site are presumably required to expand the amino acid binding pocket and result in altered substrate specificity in favor of larger ncAAs, tyrosine, and phenylalanine analogs. Substitutions in the N-domain may improve binding to tRNA_Pyl_. To our knowledge, with the exception of Asp2Asn (Bryson et al., 2017; Zhang et al., 2025), His62Tyr (Suzuki et al., 2017; Tseng et al., 2020), and Tyr306 (Yanagisawa et al., 2008), none of these residues have previously been identified or targeted for engineering in MmPylRS or other PylRS homologs.

### 3.4 MmPylRS(mut) and ChPylRS(mut) variants are active and selective for ncAAs *in vivo*

**FIGURE 5:**
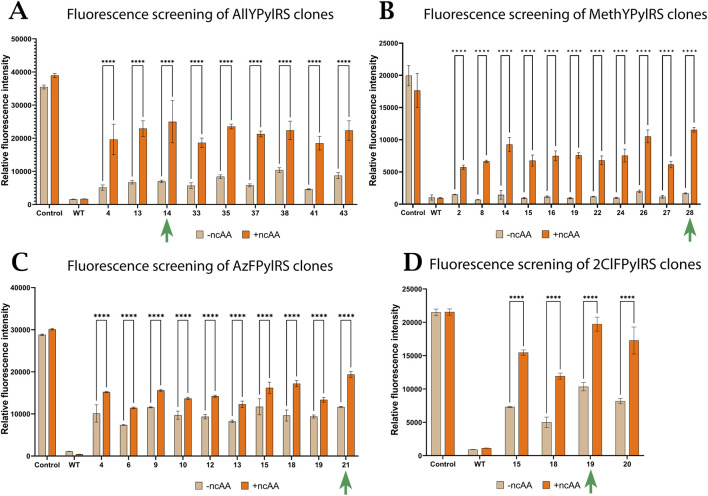
Fluorescence measurements of the most active mutants with and without the addition of **(A)** O-Allyl-L-Tyr (AllYPylRS), **(B)** O-Methyl-L-Tyr (MethYPylRS), **(C)** 4-Azido-L-Phe (AzFPylRS), and **(D)** 2-Chloro-Phe (2CloFPylRS). Positive control: cells expressing sfGFP without a stop codon. WT: cells containing the original PylRS that did not undergo PANCE (MmPylRS(mut) for O-Allyl-L-Tyr, O-Methyl-L-Tyr, and 4-Azido-L-Phe; ChPylRS(mut) for 2-Chloro-Phe). Error bars represent ±standard deviation of the mean of three independent replicates. Green arrows indicate the clones selected for further analysis.

To evaluate the efficacy of mutant aaRS forms, a fluorescence assay was performed using the sfGFP protein in the *E. coli* BL21 Star expression system. One of the test system plasmids (pET-sfGFP-27TAG) contains the green fluorescent protein gene with an amber stop codon at position 27, resulting in premature translation termination if the required aaRS for incorporating the non-canonical amino acid is absent. In addition to the pET-sfGFP-27TAG plasmid, the test system included mutant AllYPylRS, AzFPylRS, and MethYPylRS as part of the pHpyl-MmPylRS-GlnS plasmid, and 2ClFPylRS as part of the pHpyl-ChPylRS-GlnS plasmid. Thus, fluorescence from sfGFP production can be used to measure the activity of new PylRS variants. For *in vivo* fluorescence analysis, normalisation was performed to the optical density of the culture (OD600), and background subtraction was carried out for the medium with and without the addition of ncAA for the respective cultures. We measured the fluorescence/OD600 of cells expressing PylRS variants and compared it with cells expressing wild-type PylRS, both in the presence and absence of the corresponding amino acids. More than 22 clones were tested for each line, and the results for the best ones are presented in Figure 5.

The results show that each of the four mutant constructs exhibits high activity and selectivity for its corresponding ncAA. In Figure 5, the evolved synthetases differ not only in maximum fluorescence in the presence of their cognate ncAA but also in basal fluorescence in the absence of ncAA. We speculate that differences in ncAA uptake or toxicity may cause this variability. It was previously observed in the study that tyrosine and phenylalanine ncAA analogues absorb in the same spectrum as sfGFP, which may influence the differences between experiments with different amino acids. However, the main conclusion in this context is not the absolute comparison of potency between different pairs, but the demonstration that each evolved PylRS variant acquired pronounced and specific activity towards its target ncAA compared to the parent enzyme. Without the corresponding ncAA in the medium, the evolved clones showed low levels of sfGFP expression. In contrast, the addition of ncAA promoted sfGFP overexpression. Based on the results, the observed clones exhibited significant differences (p < 0.0001) in fluorescence intensity in the absence and presence of the corresponding ncAA in the medium. In all cases, the activity of the synthetases that underwent PACE was significantly higher than that of wild-type aaRS. In some clones with the most active synthetase variants, the fluorescence intensity exceeded 50% of that of the positive control. To further analyse ncAA incorporation, we selected the most efficient clones of each PylRS in the assay with the corresponding amino acid (indicated by green arrows in Figure 5).

**FIGURE 6:**
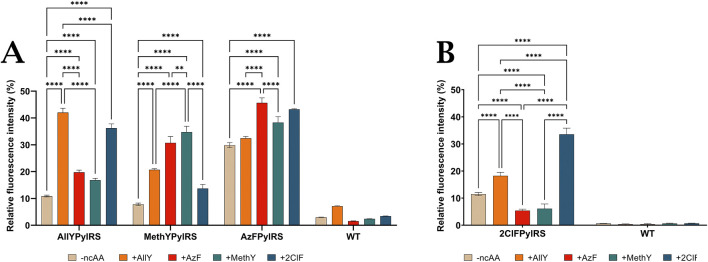
Relative fluorescence intensity of the most active mutants with and without the addition of O-Allyl-L-Tyr, O-Methyl-L-Tyr, 4-Azido-L-Phe and 2-Chloro-Phe for **(A)** AllYPylRS, MethYPylRS, AzFPylRS and **(B)** 2CloFPylRS. WT: cells containing the original PylRS that did not undergo PANCE (MmPylRS(mut) for O-Allyl-L-Tyr, O-Methyl-L-Tyr, and 4-Azido-L-Phe; ChPylRS(mut) for 2-Chloro-Phe). Error bars represent ±standard deviation of the mean of three independent replicates.

Due to the structural similarity of all four ncAAs used in this study, the cross-reactivity of the evolved PylRS to non-target substrates was examined (Figure 6). As expected, each synthetase demonstrated the highest efficiency of incorporation of its target amino acid analogue into sfGFP. However, a characteristic specificity was revealed: AllYPylRS efficiently incorporated 2ClF (36%), while 2ClFPylRS utilised AllY (18.2%), whereas the interaction of these enzymes with the remaining ncAAs was significantly weaker. Also noteworthy are the similar fluorescence levels observed for the MethY (34.7%) and AzF (30.7%) substrates in the case of MethYPylRS, indicating its ability to cross-interact with this pair of substrates. At the same time, AzFPylRS demonstrated comparable activity towards both its main substrate AzF (45.6%) and 2ClF (43.2%). The absence of a statistically significant difference in fluorescence indicates that this evolved synthetase has broad substrate specificity.

**FIGURE 7:**
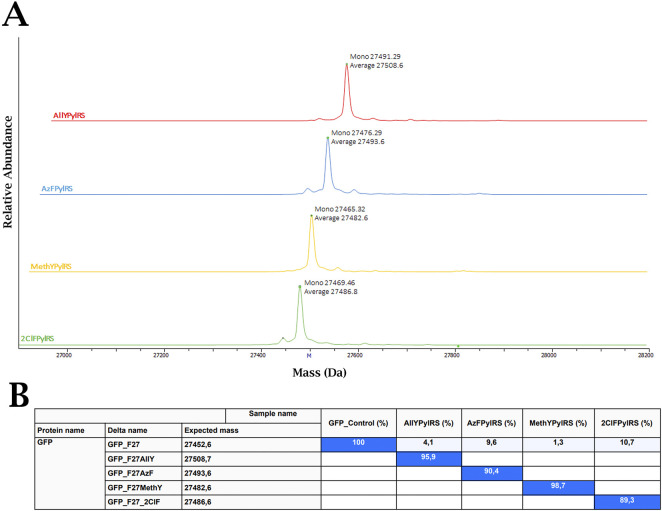
**(A)** ESI-MS spectra of sfGFP co-expressed with the corresponding PylRS in the presence of the target ncAA. **(B)** Relative signal intensity values for each protein detected and identified by mass spectrometry. The relative signal values for sfGFP containing the target ncAA are shown in blue.

To comprehensively assess the efficiency of target ncAA incorporation, purified sfGFP samples underwent mass spectrometric analysis. *E. coli* BL21 Star cells were co-transformed with the pHPyl-PylRS-GlnS plasmid, carrying the corresponding evolved PylRS gene, and pET-sfGFP-27TAG, encoding a reporter protein with a TAG stop codon at position 27. Expression was induced in the presence of 2 mM of the corresponding ncAA. Intact mass analysis confirmed the expected molecular mass of the proteins, demonstrating specific incorporation of the target analogue into the sfGFP at the target position (Figure 7A). Additionally, the proportion of full-length ncAA-containing proteins relative to the total pool of identified sfGFP-like proteins was quantified. Under all experimental conditions, the proportion of the target product exceeded 89% (Figure 7B). The most intense minor peaks in the mass spectra corresponded to the mass of the protein with incorporated phenylalanine, a characteristic artefact caused by incomplete suppression of stop codon translation and competitive incorporation of the natural amino acid.

## 4 Discussion

Currently, numerous ncAAs can be site-specifically incorporated into proteins of interest by expanding the genetic code (Mariotti et al., 2013; Gong et al., 2023; Dakhnevich et al., 2025). Site-directed incorporation of ncAAs into proteins requires appropriate cellular mechanisms. In addition to a free codon, the presence of orthogonal aaRS/tRNA pairs is important (Ding Y. et al., 2020). Engineering orthogonal aaRSs capable of highly selective ncAA incorporation remains a central challenge for genetic code expansion. The classical rational design approach, based on knowledge of the three-dimensional structure, often targets a limited set of key amino acids in the active site (Zhang et al., 2025) (Supplementary Table S3). This approach focuses on previously studied active site candidates, overlooking complex networks of distal interactions and synergistic effects that can significantly influence protein function. An alternative strategy is directed evolution (Bryson et al., 2017). Evolutionary selection can produce combinations of substitutions in different parts of the protein that collectively enhance function. Such subtle interactions would be extremely difficult to predict computationally. Directed evolution enables not only the improvement of known synthetases but also the discovery of previously unknown active site amino acid targets for rational design.

Directed evolution of enzymes *in vivo*, particularly using the PANCE method, relies on the assumption of a direct link between the function of the target protein and the selective advantage of the phage containing this enzyme (Carlson et al., 2014). During the evolution of PylRS, selection pressure is applied to its ability to acylate tRNA in the presence of ncAA. However, the effectiveness of selection is determined by the entire experimental system, including the expression levels of the selective genes (gIII and gIII-neg), the activity of T7 RNA polymerase, the availability of ncAA, and cellular physiology. Therefore, the observed evolutionary outcome is an integrated property of the whole system (Esvelt et al., 2011; Miller et al., 2020).

Critically, the genetic architecture of PANCE strictly limits the adaptability of accessory components (Miller et al., 2020). In addition to its own genes, the mutagenic phage M13 carries only the PylRS gene under its own promoter and serves as the sole target for site-directed mutagenesis. All other elements—the gIII, gIII-neg, and T7 RNA polymerase genes—are located on stable helper plasmids, which are not subject to targeted evolution as part of the protocol. Therefore, primary adaptations occur within the PylRS gene itself. However, preset system parameters (e.g., the balance of gIII and gIII-neg expression) shape the selective landscape, determining the stringency of selection. Incorrectly setting these parameters, for example, through mutations in the promoter regions of genes on helper plasmids, can bias evolutionary trajectories, such as favouring the selection of variants with increased basal activity rather than those with improved ncAA specificity. Random mutations that increase a cell’s overall tolerance to stress associated with protein overexpression or the presence of ncAAs may weaken the overall selection pressure, allowing suboptimal PylRS variants to become established. However, this remains an unlikely scenario.

In the present study, a set of measures was implemented to minimise system artefacts and ensure that evolution was directed exclusively towards the target gene. To prevent the accumulation of adaptive mutations in the host cell genome and auxiliary plasmid constructs, regular cell population rotation was employed: the phage pool was transferred daily to a fresh cell culture (Suzuki et al., 2017). This procedure effectively eliminated potential cellular mutants. Mutagenesis was induced only upon infection of a fresh cell pool with phage libraries, preventing the preliminary accumulation of random mutations in the regulatory elements of the system and ensuring targeted variability.

We also demonstrated that the PANCE system can be optimised to produce more specific PylRS by integrating a negative selection step into the procedure. The proposed negative selection scheme, aimed at suppressing activity against competing substrates in the absence of the target ncAA, is a critical component for evolving strict specificity, which often presents the greatest challenge in PylRS design. In our work, we developed a negative selection process for PANCE based on previously reported variants (Carlson et al., 2014) designed to specifically suppress the activity of mutant enzymes lacking the required specificity. Our strategy was based on the assumption that under conditions of T7 RNA polymerase activation, when PylRS does not recognise ncAA but instead incorporates natural amino acids in place of the amber stop codon in the T7RNAP transcript, the level of gIII-neg production is sufficient to dominantly inhibit infectious phage assembly.

However, an intermediate selection scenario remains possible. When gIII-neg expression is suboptimal or incomplete, phage particles carrying PylRS variants with undesirable, but not maximal, basal activity for canonical amino acids may escape negative selection. To minimise this risk, our experimental strategy was designed with multiple levels of control. Therefore, we conducted not one, but several sequential rounds of negative selection, significantly increasing the likelihood of eliminating particles that escaped due to a temporary or quantitative deficiency of gIII-neg in individual cells. A key element of the protocol was the mandatory testing of the effectiveness of these sequential rounds of negative selection by comparing phage titres after a round of appropriate stringency without mutagenesis on cells in the presence and absence of ncAA. We also confirmed differences in titres of several orders of magnitude after rounds of negative selection (Supplementary Figure S1). An insufficient difference was considered an indicator of potential escape and served as the basis for returning to the previous stage of evolution and repeating the selection. An important part of the study was not only *in vivo* selection, but also subsequent testing of PylRS variants and quantitative assessment of their activity in a fluorescence selection system and by mass spectrometric analysis of ncAA incorporation into sfGFP, which completely eliminated the influence of cellular context, including the pIII/gIII-neg balance. Although the quantitative parameters of the negative selection system in our study were not calibrated, the iterative feedback strategy used, with mandatory *in vitro* validation, was specifically designed to ensure high selection stringency and minimise the likelihood of enrichment of ncAA-independent variants. Within this approach, multi-step validation successfully compensated for the uncertainty in pIII/gIII-neg expression levels.

Our work on phage evolution produced a large pool of PylRS mutant variants, which we analysed. High-throughput sequencing on the MiSeq platform enabled us to identify major mutational lineages established through targeted selection (Supplementary Table S2). Further screening with a fluorescent reporter assay identified the best clones, which contained both conservative and novel, previously unnoticed minor mutations that determine the improved functional properties of PylRS (Figure 4).

**FIGURE 8:**
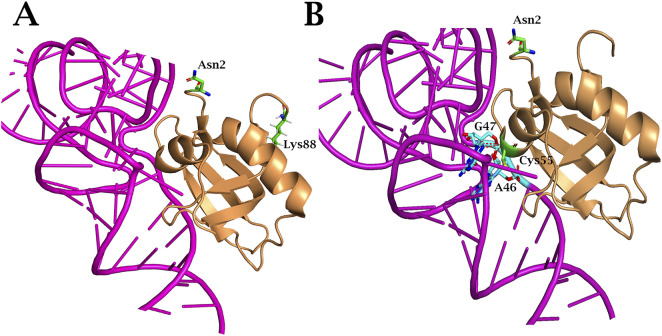
Structure of MmPylRS NTD complex with tRNA_Pyl_ of **(A)** AllPylRS and **(B)** AzFPylRS. Amino acid substitutions that occurred during PANCE are indicated in green. The red dashed lines represent potential hydrogen-bonding interactions. Such interactions are illustrated between the side chain of Arg55 within AzFPylRS NTD and the phosphodiester backbone of A46 and G47 in tRNA_Pyl_. The structure is based on the PDB entry 5V6X.

Thus, the evolution of PylRS with various non-canonical substrates has resulted in the accumulation of multiple mutations in the N-terminal domain. Convergent substitutions are observed: AllPylRS (Figure 8A) and AzFPylRS (Figure 8B) share the same mutation, Asp2Asn, which, according to literature, significantly enhances the enzyme’s catalytic activity. This mutation has been shown to increase PylRS activity towards 3BrF threefold (Bryson et al., 2017; Zhang et al., 2025). The mechanism is associated with the disruption of hydrogen bonds between the synthetase and the T-loop of tRNA_Pyl_, allowing the formation of alternative contacts, such as Glu88Lys and Arg55Cys, identified in AllYPylRS and AzFPylRS, respectively. The mutation at Glu88 has not been previously described in the literature, while Arg55 is known to be involved in hydrogen bonding with nucleotide A46 of tRNA (Zhang et al., 2025), and substitution to cysteine may provide a stronger covalent or coordination interaction (Figure 8B).

**FIGURE 9:**
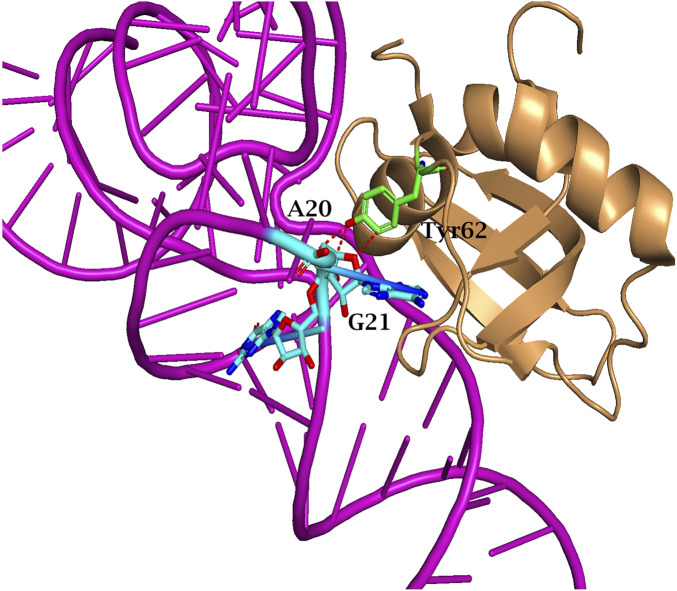
Structure of MmPylRS NTD complex with tRNA_Pyl_ of 2ClFPylRS. Amino acid substitutions that occurred during PANCE are indicated in green. The red dashed lines represent potential hydrogen-bonding interactions. Such interactions are illustrated between the side chain of Tyr62 within 2ClFPylRS NTD and the phosphodiester backbone of A20 and G21 in tRNA_Pyl_. The structure is based on the PDB entry 5V6X.

In the case of 2ClFPylRS, a His62Tyr mutation, previously described for chPylRS, was detected, which increased the efficiency of amber stop codon suppression (Bryson et al., 2017; Tseng et al., 2020). This mutation disrupts two hydrogen bonds between PylRS and the T-loop fragments of tRNA_Pyl_, although this is partially compensated by weak interactions with the G21 phosphate and A20 base (Figure 9) (Suzuki et al., 2017). Additional interactions of tRNA with the CTD of chPylRS appear to be enhanced by the Asn202Ser and Glu210Lys mutations (corresponding to Asn241 and Glu249 in *M. mazei*). Although these amino acid substitutions have not previously been described in the literature, their role may relate to enzyme stabilisation. Specifically, the Glu210Lys mutation affects the highly conserved MOTIF 1, a canonical structural element characteristic of class II aminoacyl-tRNA synthetases (Eriani et al., 1990). According to the literature, MOTIF 1 plays a key role in the dimerisation of the NTD and CTD, which is critical for the formation of a functional enzyme capable of coordinating ATP, tRNA, and ncAA molecules (Kavran et al., 2007). The Glu210Lys substitution likely leads to the formation of additional hydrogen bonds, which in turn may enhance contacts with tRNA_Pyl_ by altering the conformational dynamics of the domains (Figure 9).

**FIGURE 10:**
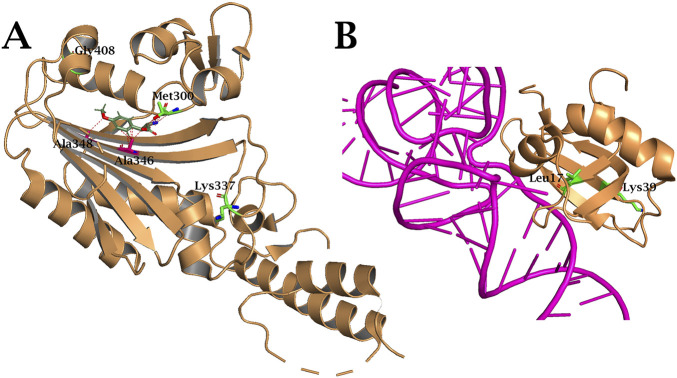
Structure of MethYPylRS **(A)** CTD and **(B)** NTD complex with tRNA_Pyl_. Amino acid substitutions that occurred during PANCE are indicated in green. The structures are based on the PDB entry 2ZCE and 5V6X.

The Met17Leu and Glu39Lys mutations, which have become entrenched in MethoYPylRS during evolution, were not previously considered as targets for rational design of the NTD of PylRS (Figure 10B). It can be hypothesised that their mechanism of action is similar to those described previously: the Met17Leu mutation likely increases the stability of the protein structure, while GluIts CTD was shown to form anextensive network of contacts with39Lys redistributes electron density, facilitating a shift in interactions with tRNA_Pyl_ from the NTD to the CTD of the synthetase. The Glu337Lys mutation in MethoYPylRS also supports the interaction redistribution hypothesis (Figure 10A). This substitution is localised in the CTD in the highly conserved MOTIF 2, which is responsible for ATP and tRNA binding (Kavran et al., 2007). A similar mechanism of tRNA binding by the CTD was previously characterised for DhPylRS from Desulfitobacterium hafniense (Nozawa et al., 2009). This synthetase, which belongs to the “PylSn + PylSc” class, exhibits significant activity *in vivo* even in the absence of a complete NTD RNA-binding domain (Fladischer et al., 2019). Its CTD was shown to form an extensive network of contacts with tRNA, including key interactions at Lys166:C72 and His168:C74 (corresponding to Lys366 and His368 in *M. mazei* PylRS) binding tRNA near adenosine 76, which is crucial for aminoacylation (Nozawa et al., 2009). Thus, the Glu337Lys mutation in MethoYPylRS appears to be designed to establish similarly strong contacts with tRNA_Pyl_ from the CTD side.

Taken together, these data suggest that the selected changes in the NTD reduce this domain’s affinity for tRNA, but this weakening of binding is likely adaptive. It promotes a redistribution of interactions with tRNA towards the CTD, ultimately leading to the formation of a more robust and specific PylRS-tRNA complex and increased efficiency of aminoacylation with new substrates.

**FIGURE 11:**
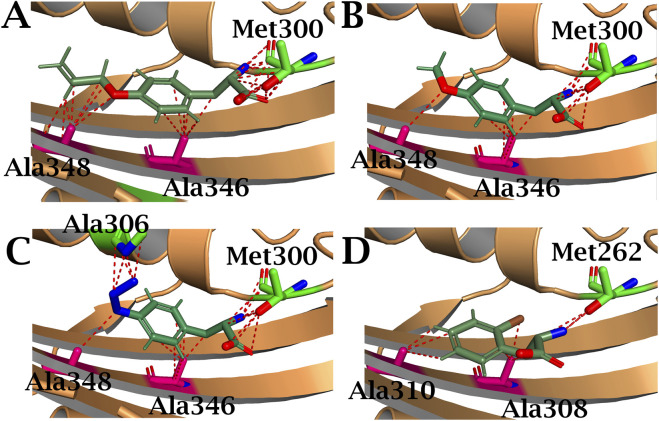
Structure of **(A)** AllYPylRS, **(B)** MethYPylRS, **(C)** AzFPylRS and **(D)** 2ClFPylRS amino acid binding pocket in complex with the corresponding ncAA. Amino acid substitutions are indicated in green. The red dashed lines represent potential hydrogen-bonding interactions. The structures are based on the PDB entry 2ZCE.

In addition to the mutations found in the N-domain, a large number of substitutions, as expected, were concentrated in the active site of PylRS, resulting in an expanded amino acid pocket for aminoacylation of tyrosine and phenylalanine derivatives. Of particular interest is the Met300Thr mutation, which arose independently in all studied enzymes and has not previously been described in the literature. Residues Met300 and Ala302 are known to be located near the α-amino group of Pyl in the catalytic core of PylRS, but their side chains are too distant from the α-amino group of Pyl to form stable van der Waals interactions. Therefore, PylRS recognises the α-amino group of the substrate indirectly, through a hydrogen bond network (Kobayashi et al., 2009; Wan et al., 2014). The Ala302Thr mutation is known to result in the appearance of a hydroxyl group that forms a protrusion near the β-sheets of the active site, defining the base of the amino acid binding site. In particular, residue Thr302 can directly form a hydrogen bond with the α-carboxyl group of the substrate without the participation of a water molecule (Takimoto et al., 2011). Based on this and 3D modelling of the Met300Thr mutation (Figure 11), it can be hypothesised that the Met300Thr mutation may similarly enhance interactions with tyrosine and phenylalanine derivatives containing a benzene ring. The hydroxyl group of threonine, forming a steric bulge in the active site, can redistribute electron density by forming hydrogen bonds with the carboxyl group of the new amino acid, thereby ensuring more efficient binding of the new substrate. As this mutation has not been previously described in the literature, its exact impact of Met300 will be the subject of future work by our group.

**FIGURE 12:**
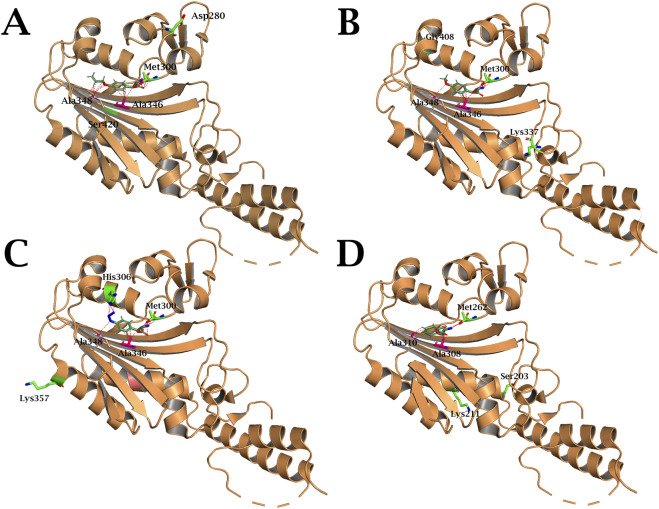
Structure of **(A)** AllYPylRS, **(B)** MethYPylRS, **(C)** AzFPylRS and **(D)** 2ClFPylRS CTD in complex with the corresponding ncAA. Amino acid substitutions are indicated in green. The red dashed lines represent potential hydrogen-bonding interactions. The structures are based on the PDB entry 2ZCE.

Furthermore, mutations identified in the CTD of PylRS are of considerable interest: Ala107Thr, Asn280Asp and Asn420Ser in AllYPylRS, Asp408Gly in MethYPylRS, and Tyr306His and Glu357Lys in AzFPylRS. These mutations appear to stabilise the enzyme’s tertiary structure and enlarge the binding pocket (Figure 12). This structural alteration optimises the enzyme for aminoacylation of tRNA with large non-cyclic amino acids, particularly phenylalanine and tyrosine derivatives with bulky substituents. Notably, among all the mutations, only one homologous residue, Tyr306, has previously been described in the literature. The Tyr306Ala substitution has been shown to significantly increase the size of the PylRS binding pocket, thereby enhancing the efficiency of ncAA incorporation *in vivo* (Yanagisawa et al., 2008; Schloßhauer et al., 2023). Based on literature data and 3D modelling (Figure 11C), it can be hypothesised that this mutation also expands the binding pocket and establishes weak hydrogen interactions with the azido group of AzF.

Fluorescence analysis showed that PylRS variants generated using PANCE efficiently incorporate four target ncAAs into protein, with the best variants exceeding 50% of the native sfGFP level and achieving more than ten times the efficiency of the original synthetase. This result is high and comparable to data from other studies using sfGFP-based assays (Odoi et al., 2013; Jiang et al., 2020). Modern *in vivo* directed evolution methodologies, including PANCE, aim for maximum specificity; however, achieving absolute (100%) ncAA dependence in living cells remains challenging. Residual background levels are linked to complex cellular physiology, including competitive translation processes. However, the proven key role of iterative negative selection in our design was significant, as this approach minimised unwanted background. In contrast, protocols lacking strict negative selection often enrich variants with high basal activity but without the required ncAA dependence (Supplementary Table S3). Mass spectrometric analysis shows that the incorporation efficiency of the target ncAAs into sfGFP is 89%, which is suitable for most applications. Thus, the orthogonal PylRS variants developed in this study, as well as the *E. coli* strains containing them, offer opportunities for cost-effective biosynthesis of proteins and peptides containing non-proteinogenic amino acids.

In particular, the incorporation of azide- and halogen-containing n-acetyl amino acids represents a bioorthogonal and biocompatible approach to protein modification, for example, using SPAAC. This approach has significant potential in medical biotechnology for both basic research (*in situ*) and applied *in vitro* studies aimed at creating new therapeutic and diagnostic molecules (Arpino et al., 2015). Previously described mutant PylRS from *Methanosarcina mazei*, used to genetically encode tyrosine and phenylalanine analogues, are reported in the literature. The Y306A/Y384F variant (Tookmanian et al., 2014; Yanagisawa et al., 2019) is widely used for AzF incorporation, and the N7Y/H63L/K67N/V74W mutant was designed for specific acylation of tRNA_Pyl_ 2ClF (Zhang et al., 2025). The PylRS variants we developed differ from these analogues by a fundamentally new set of amino acid substitutions, resulting from a different methodological approach to their creation. Traditional strategies, such as rational design aimed at modifying the amino acid binding pocket or directed evolution focusing on this region, often do not consider possible cooperative rearrangements in the enzyme structure. In contrast, the strategy we used potentially allows for the inclusion of such effects, which could lead not only to a new mutation pattern but also to improved catalytic properties. Fluorescence analysis of the efficiency of our variant for incorporating 2ClF revealed an approximately 34-fold activity rate compared to the wild-type enzyme, which is quantitatively comparable to the 28.4-fold improvement reported for the mutant (Zhang et al., 2025). However, direct and unambiguous comparison of these numbers is difficult, as they were obtained in different experimental systems. Therefore, our reported efficiency, although encouraging, requires direct validation under identical conditions.

The primary goal of our study is not only to create new tools for specific ncAAs, but also to demonstrate the fundamental potential of the PANCE method for identifying new, often non-obvious, mutations in PylRS and redirecting its substrate specificity. In this study, a multi-stage scheme for the directed evolution of pyrrolysine synthetases using the PANCE method was successfully implemented and optimised. Positive selection involved serial passages of the phage in a medium containing the target ncAA, with stepwise tightening of the selection conditions for the mutated synthetase. Host cells expressed the mutant aminoacyl-tRNA synthetase, and the phage survived and replicated only if its genome contained a functional gene pair (aaRS/tRNA), enabling utilisation of the ncAA for protein assembly. A key innovation was the development of a negative selection protocol: after each stage of positive selection, targeted rounds of selection were conducted in the absence of ncAA, ensuring effective suppression of nonspecific or “conditionally active” mutants capable of functioning without the target substrate. This iterative approach, alternating phases of target function enhancement and elimination of undesirable variants, greatly increased the selective pressure in favour of synthetases with true dependence of activity on the presence of ncAA. A quantitative criterion for selecting functional enzyme variants was also crucial, based on comparison of the titres of the bacteriophage carrying the synthetase gene in the presence and absence of the target ncAA in the medium. Progression to the next, more stringent stage of positive selection was permitted only when a titre difference of 1.5–2 orders of magnitude was achieved, indicating the high specificity and functionality of the selected mutants *in vivo*. The system demonstrates high stability; however, as shown by the evolution of the synthetase for AzFPylRS, in some cases a strategic return to less stringent conditions is required to overcome evolutionary barriers. The entire process was controlled through standardised parameters—culture optical density, cultivation time, and PFU level—enabling reproducible and controlled selection of variants with improved catalytic properties and strict substrate specificity. The key novelty lies in the methodology itself and in the catalogue of mutations, such as Met300Thr, which have not previously been considered as targets for rational design. The results of aaRS evolution and screening indicate that interactions between the ncAA substrate and the enzyme can be enhanced by multiple mutations that establish and fine-tune selectivity and activity. This opens new avenues for in-depth study of PylRS and provides a foundation for other substrates.

## Data Availability

The original contributions presented in the study are publicly available. This data can be found here: https://www.ncbi.nlm.nih.gov/bioproject/PRJNA1428242.
