# Supplementary material for: Expanding the genetic code: phage-driven evolution of pyrrolysyl-synthetase for site-specific incorporation of synthetic phenylalanine and tyrosine derivatives
Source: Front Mol Biosci. 2026 Mar 17;13:1737987. doi: 10.3389/fmolb.2026.1737987 (PMC13035772; doi:10.3389/fmolb.2026.1737987)
Supplement: Supplementary file 1 [file DataSheet1.pdf]

Supplementary Material

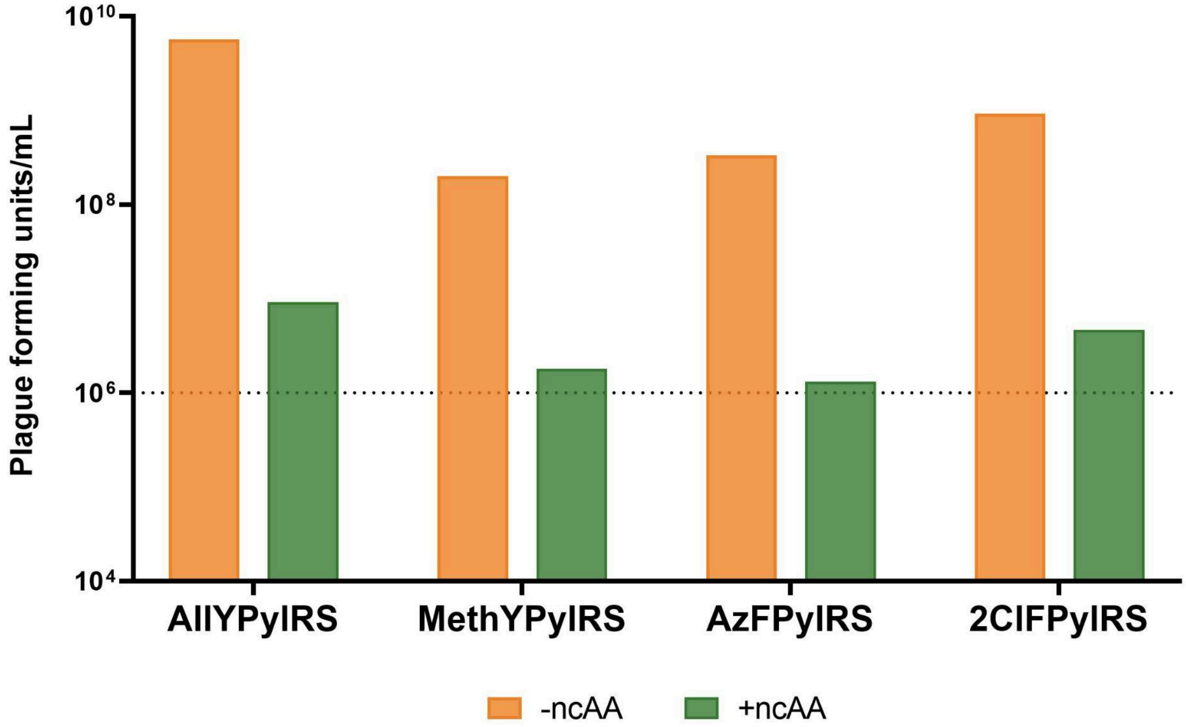

Supplementary Figure 1 – Measurements of phage titers comparing evolved phages in the presence and absence of ncAA. Titers are individual measurements based on the average of three technical replicates of the qPCR assay.

Supplementary Material

Supplementary Table 1 – Plasmid constructs used in this work

Name	Antibiotic Resistance	Origin	ORIF1		ORIF2	
			Promoter	Gene	Promoter	Gene
pMP6C	$chlor^R$	cloDF13	P_{AraBAD}	dnaQ926, dam, seqA, emrR, ugi, cda1, dam		
pDB021CH(+)	amp^R	pSC101	P_{T7}	gIII, luxAB	P_{proK}	$tRNA^{Pyl}$
pDB023f	spc^R	ColE1	Ppsp	T7RNAP+ 2 amber codons		
pDB038	spc^R	ColE1	P_{psp}	gIII(P29*)	P_{proK}	$tRNA^{Pyl}$
pDB038a	spc^R	ColE1	P_{psp}	gIII(P29*, Y184*), luxAB	P_{proK}	$tRNA^{Pyl}$
pDB038b	spc^R	ColE1	P_{psp}	gIII(P29*, P83*, Y184*), luxAB	P_{proK}	$tRNA^{Pyl}$
pDB016-$tRNA_{Pyl}$	spc^R	ColE1	P_{T7}	gIII-neg	-	-
pDB007ns2a neg	amp^R	pSC101	P_{psp}	gIII	P_{psp}	T7-RNAP
pHpyl-MmPylRS-GlnS	$chlor^R$	P15A	GlnS	MmPylRS _mut	P_{proK}	$tRNA^{Pyl}$
pHpyl-ChPylRS-GlnS	$chlor^R$	P15A	GlnS	chPylRS	P_{proK}	$tRNA^{Pyl}$

pET-sfGFP-27TAG	kan^R	pUC	P_{T7}	Superfolder GFP (F27*)	-	-
SP-MmPylRS	none	M13 f1	P_{gIII}	Mm PylRS		
SP-chPylRS mut	none	M13 f1	P_{gIII}	ch PylRS		

Supplementary Table 2 - Mutations identified by MiSeq and their frequency in PylRS genes from different stages of PANCE

AllYPylRS						
Nucleotide position	Nucleotide substitution	Amino acid substitution	Frequency Stage 1	Frequency Stage 2	Frequency Stage 3	Frequency Stage 4
4	G => A	Asp2Asn	0,7010	0,9996	0,9995	0,9990
262	G => A	Glu88Lys				0,9200
549	G => A	Val183Val		0,4363	0,6933	0,7663
899	T => C	Met300Thr	0,6347	0,7089	0,8255	0,6886
MethYPylRS						
Nucleotide position	Nucleotide substitution	Amino acid substitution	Frequency Stage 1	Frequency Stage 2	Frequency Stage 3	Frequency Stage 4
192	G => A	Met17Leu			0,9986	0,9986
899	T => C	Met300Thr	0,7921	0,7182	0,8315	0,7150
1009	G => A	Glu337Lys			0,9941	0,9985
1223	A => G	Asp408Gly	0,5076	0,9991	0,9988	0,9989
AzFPylRS						
Nucleotide position	Nucleotide substitution	Amino acid substitution	Frequency Stage 1	Frequency Stage 2	Frequency Stage 3	Frequency Stage 4
4	G => A	Asp2Asn			0,9990	0,9979
750	C => T	Ile250Ile				0,8659
899	T => C	Met300Thr	0,8383	0,7344	0,8249	0,4588
916	T => C	Tyr306His		0,1631	0,9979	0,9982
1069	G => A	Glu357Lys				0,4403
2ClFPylRS						
Nucleotide position	Nucleotide substitution	Amino acid substitution	Frequency Stage 1	Frequency Stage 2	Frequency Stage 3	Frequency Stage 4
184	C => T	His62Tyr	0,1651	0,3816	0,9616	0,9975
623	A => G	Asn100Ser		0,1274	0,0763	0,9660
800	T => C	Met159Thr	0,8122	0,9989	0,9986	0,9989

Table 3. Comparison of early major studies on mutagenesis and directed evolutionии

Study	aaRS/ PylRS	Selection format	Experimental design	ncAA	Results
DOI: 10.1073/pnas.1419737111	PylRS variants (AcKRS, IFRS, etc.)	Plasmid in vivo selection (positive + negative rounds)	Rounds of positive and negative selection.<br>Positive selection involves translation of the TAG codon at position 112 in chloramphenicol acetyltransferase in the presence of 1 mM 3-I-Phe (expression plasmid pCAT-pylT). The selected library is then subjected to negative selection by translating the TAG codons at positions 13 and 44 in the toxic protein ccdB in the absence of 3-I-Phe (expression plasmid pAraCB2-pylT).<br>Positive selection identifies tRNA synthetase variants that translate UAG using ncAA and/or canonical amino acids. Negative selection eliminates variants that favour the incorporation of canonical amino acids.	Phenylalanine derivatives (3-I-Phe, 3-Br-Phe, 3-Cl-Phe, 3-Me-Phe, etc.)	Polyspecificity, 30-fold increase in efficiency when working with phenylalanine derivatives.
DOI: 10.1038/nchembio.2474	Chimeric pyrrolysyl-tRNA synthetase from Methanosarcina spp.	PACE	Dual positive and negative selection (PACE).<br>Host cells were initially cotransformed with plasmids pDB021CH(+), pDB023f, and DP4. To further enhance selection, pDB038, pDB038a, and pDB038b (plasmids with the gIII gene containing one, two, or three amber stop codons, respectively) were used. Three host cell strains were used for dual selection: host A (pDB007(+), pDB023f1, and DP4); host B (pDB007(+), pDB023f1, and MP4); and host C (pDB007(+)ns2a, pDB016, and MP4).	p-Nitrophenylala<br>Nε-Boc-lysine<br>Nε-Acetyllysine<br>m-Iodophenylalanine<br>p-Iodophenylalanine	Enzymatic efficiency increased 45-fold compared to the parent enzyme. The yield of proteins containing non-canonical residues increased by up to 9.7-fold.
doi.org/10.1038/s41598	PylRS Methanosarcina	Fuorescence-activated	Initially, a library of PylRS mutants was generated, selecting six positions (Y271, L274, C313, M315,	31 structurally diverse ncAAs	Prediction and generation of a

-019-48357-0	barkeri (M. barkeri)	cell sorting (FACS) based screening method	V370, and I378) based on structural considerations. For FACS screening, the PylRS library, tRNAPyl, and cyan monomeric turquoise fluorescent protein 1 (mTFP1) were expressed from a single plasmid (EVOL303_Lib). mTFP1 contained an amber codon at position 128 (mTFP1TAG128). *E. coli* cells transformed with pEVOL303_Lib were grown in both the absence and presence of noncanonical amino acids. Key mutations (Y271A, L274V, C313V, M315Y, Y349F, and V370R) were combined to create a highly polyspecific PylRS.	bearing clickable, fluorinated, fluorescent, and biotinylated entities	highly polyspecific pyrrolysyl-tRNA synthetase (HpRS) for the incorporation of large non-canonical amino acids.
DOI: 10.3389/fmolb.2022.850613	PylRS from Methanomethylophilus alvus (Ma PylRS)	PANCE	Positive selection was carried out using E. coli S1030 cells transformed with the helper plasmid pJT017 (gIII1xTAG MatRNAPyl) and the mutagenic plasmid MP4 during rounds of mutagenesis. In subsequent, more stringent rounds of positive selection, plasmids pJT018 (gIII2xTAG MatRNAPyl) and pJT019 (gIII3xTAG MatRNAPyl) were used instead of pJT017. Positive selection was performed with the addition of 5 mM BocK NPA.<br>Negative selection was carried out using the negative selection plasmids pJF011 (T7RNAP2xTAG MatRNAPyl(6)) and pDB016 (carrying gIII under the T7 promoter). No NPA was added.	Nε-Boc-l-lysine (BocK)<br><br>Additional ncAAs: Nε-propargyloxycarbonyl-l-lysine (PrK), Nε-allyloxycarbonyl-l-lysine (ALocK), 4-azido-l-phenylalanine (pAzF), and 3-iodo-l-phenylalanine (mIF)	A variant of the PylRS_opt enzyme was developed. PylRS_opt excludes canonical amino acids and recognises a broad range of NPAs; when using the amino acids BocK, ALocK, and PrK in a fluorescence assay, PylRS_opt activity is significantly higher than that of MaPylRS.<br>Using PylRS_opt results in more than a twofold improvement in protein yield

					compared to wild-type MaPylRS.
DOI: 10.1021/acsbiomedchemau.4c00028	PylRS from Candidatus Methanomethylophilus alvus (CmaPylRS) + CMatRNAPyl-C41AU (C41AU-mutant tRNA^Pyl)	Directed evolution by randomizing its coding sequence, followed by the screening of active mutant clones	To enhance the reactivity of CmaPylRS towards CmatRNA Pyl-C41AU, directed evolution was performed using two plasmids. The first, pBK-CmaPylRS, encodes the CmaPylRS gene, which was subjected to randomisation and is under the control of the glutamine synthetase (GlnS) promoter. The second plasmid, pY+-CmatRNA Pyl-C41AU, encodes the CmatRNA Pyl-C41AU gene, the chloramphenicol acetyltransferase (ChlR) gene with two amber mutations at coding positions N2 and D44, the T7 RNA polymerase (T7RNAP) gene with two amber mutations at coding positions M1 and Q107 and the MTMITVH lead peptide, as well as the ultraviolet-excitable GFP (GFP UV) gene under the control of the T7/Lac promoter. The ChlR gene enables survival in the presence of chloramphenicol only for cells with strong suppression of the amber mutation. The T7RNAP and GFP UV genes function together, allowing cells with strong suppression of the amber mutation to exhibit strong green fluorescence under UV light. Randomisation was performed using GeneMorph II. The best selected clone, R1-6, was then used for a second round of mutagenesis and screening. The pBK-R1-6 plasmid was first sequence-randomised using the GeneMorph II kit. The resulting plasmid library was then used to transform E. coli Top10 cells containing the pY+-CmatRNA Pyl-C41AU plasmid. The cells were grown in medium supplemented with BocK. The results identified a new best clone, R2-7. In the third	Nε-tert-butyloxycarbonyl-l-lysine (BocK) and Nε-acetyl-l-lysine (AcK)	Enhancement of the catalytic efficiency of CmaPylRS towards CmatRNAPyl-C41AU (new clone R3–14).

			round, the resulting randomised R2-7 DNA was cloned into the pEVOL template. The resulting pEVOL plasmid library was used to transform Top10 E. coli cells containing the pBAD-sfGFP134TAG plasmid. The transformed cells were then plated on BocK-containing plates. Clones A, R3-9, and R3-14 showed a clear improvement in BocK incorporation at the amber codon. Two plasmids were used to test the genetic incorporation of BocK and AcK into the amber and ochre codons, respectively. The first plasmid is a pEVOL vector containing genes encoding MmAcKRS1 and MmtRNA UUA Pyl for ochre suppression. The second plasmid is based on the pBAD vector, into which the sfGFP gene was introduced, containing a TAG mutation at M1, a TAA mutation at D134, and an additional N-terminal Met-Ala. Genes encoding wild-type CmaPyl Pyl-C41AU and CmaPylRS, R3-9 or R3-14 were also cloned into this pBAD vector.		
https://doi.org/10.1016/j.chembiol.2008.10.004	PylRS M. mazei	Multistep Engineering	The PylRS–tRNAPyl pair was evolved by random screening to increase productivity with BocLys and AlocLys. The PylRS gene was expressed under the control of the E. coli TyrRS promoter and terminator in the pTK2-1 plasmid (a derivative of pACYC184, but with kanamycin resistance and a single copy of the tRNAPyl gene under the control of the E. coli lpp promoter). The PylRS gene was randomly mutagenised using the GeneMorph PCR mutagenesis kit (Stratagene) and ligated back into pTK2-1 to generate a PylRS library. The mutant PylRS library was first subjected to positive selection based on suppression of an amber stop codon at a non-essential	Nε-(o-azidobenzyloxycarbonyl)-L-lysine (AzZLys)<br><br>Nε-(tert-butyloxycarbonyl)-L-lysine (BocLys)<br><br>Nε-allyloxycarbonyl-L-lysine (AlocLys)<br><br>*N*ε-benzyloxycar	PylRS with mutations Y306A and Y384F enabled the large-scale production of proteins specifically containing AzZLys (>10 mg per litre of medium).

			position in the chloramphenicol acetyltransferase (CAT) gene. Cells transformed with the PylRS mutant library and the wild-type tRNAPyl gene were grown in medium containing 1 mM BocLys and tested for viability in the presence of various concentrations of chloramphenicol. The PylRS(Y384F) mutant showed increased activity for BocLys, AlocLys, and pyrrolysine. A PylRS(Y306A) mutant was then developed to significantly increase the activity of aminoacylated ZLys. The double mutant PylRS(Y306A•Y384F) was effective in inhibiting AzZLys function.<br>Amber suppression and Western blot analysis: E. coli cells containing the GST(25TAG), PylRS, and tRNAPyl genes were grown in LB medium or modified M9 minimal medium, with or without added amino acids, after which protein expression was induced with IPTG. Cell extracts were fractionated by 15% SDS-PAGE and transferred to an Immobilon P membrane (Millipore). GST was detected using an anti-GST antibody (GE Healthcare) and visualised with Immobilon Western blotting reagents (Millipore).	bonyl-L-lysine (ZLys)	
Current work	PylRS from Methanosarcina mazei and chimeric construct (ChPylRS) based on domains from Methanosarcina barkeri and	PANCE	For the first stage of PACE, we used the pDB023f plasmid with two amber stop codons in the T7 RNA gene and pDB021CH(+) with the gIII gene under the T7 promoter. For subsequent stages, we used plasmids with the gIII gene containing one or more amber stop codons (pDB038, pDB038a, pDB038b). For mutagenesis with positive selection, we used the pMP6C (MP) plasmid with six mutagenesis factors. Negative selection was achieved using the	O-methyl-L-tyros ine,<br>4-azido-L-phenyl alanin,<br>O-allyl-L-tyrosine ,<br>2-chloro-L-pheny lalanine	Several new, specific APC variants were obtained. In all cases, the percentage of protein containing the target unnatural amino acid was

	Methanosarcina mazei		pDB016+tRNAPyl plasmid, which contains the gIII-neg gene with a deletion under the T7 promoter. We also used the pDB007nsa2-neg plasmid, which contains the gIII gene without stop codons under the phage shock promoter, and T7 RNA polymerase with stop codons.		greater than 89%.
